# SUPPORT NEEDS AND RESOURCES OF OLDER WOMAN LIVING WITH HIV

**DOI:** 10.1093/geroni/igac059.1045

**Published:** 2022-12-20

**Authors:** Jasmine Manalel, Jennifer Kaufman, Mark Brennan-Ing

**Affiliations:** Hunter College, New York, New York, United States; Brookdale Center for Healthy Aging Hunter College, CUNY, New York, New York, United States; Hunter College, New York, New York, United States

## Abstract

People living with HIV are at increased risk for social isolation and sparse support networks, making it difficult to navigate complex caregiving needs as they age. This study aimed to identify the support needs and resources of older women living with HIV, an understudied population in this field, and their association with well-being. Participants included 217 heterosexual women aged 50 to 77 (Mage=59) from the ROAH 2.0 Study. Almost half of the sample (47.7%) had never required assistance because of HIV or other illness or disability, whereas the remainder required current or past assistance. Preliminary findings controlling for sociodemographics indicated that availability of emotional support, but not practical support (i.e., having someone to count on for activities of daily living), was associated with lower depressive symptomology. Identifying sources and types of support will help older women leverage existing social ties or form new ones to promote successful aging with HIV.

